# Predictive value of serum MED1 and PGC-1α for bronchopulmonary dysplasia in preterm infants

**DOI:** 10.1186/s12890-024-03145-z

**Published:** 2024-07-28

**Authors:** Mengzhao Li, Wenqiang Sun, Changchang Fu, Shuyang Xu, Chengzhu Wang, Huijuan Chen, Xueping Zhu

**Affiliations:** 1 Department of Neonatology, Children’s Hospital of Soochow University, Suzhou, China; 2 Department of Child and Adolescent Healthcare, Children’s Hospital of Soochow University, Suzhou, China

**Keywords:** Bronchopulmonary dysplasia, Mediator complex subunit 1, Peroxisome proliferator-activated receptor gamma coactivator-1alpha, Predictive value

## Abstract

**Objective:**

This study aimed to predict the bronchopulmonary dysplasia (BPD) in preterm infants with a gestational age(GA) < 32 weeks utilizing clinical data, serum mediator complex subunit 1 (MED1), and serum peroxisome proliferator-activated receptor gamma coactivator-1alpha (PGC-1α).

**Methods:**

This prospective observational study enrolled 70 preterm infants with GA < 32 weeks. The infants were categorized into two groups: non-BPD group(*N* = 35) and BPD group(*N* = 35), including 25 cases with mild BPD and 10 patients with moderate/severe subgroups. We performed multifactorial regression analysis to investigate the postnatal risk factors for BPD. Furthermore, we compared serum levels of biomarkers, including MED1 and PGC-1α, among infants with and without BPD at postnatal days 1, 7, 14, 28, and PMA 36 weeks. A logistic regression model was constructed to predict BPD’s likelihood using clinical risk factors and serum biomarkers.

**Results:**

Serum levels of MED1 on the first postnatal day, PGC-1α on the 1st, 7th, and 28th days, and PMA at 36 weeks were significantly lower in the BPD group than in the non-BPD group (*P* < 0.05). Furthermore, the predictive model for BPD was created by combing serum levels of MED1 and PGC-1α on postnatal day 1 along with clinical risk factors such as frequent apnea, mechanical ventilation time > 7 d, and time to reach total enteral nutrition. Our predictive model had a high predictive accuracy(C statistics of 0.989) .

**Conclusion:**

MED1and PGC-1α could potentially serve as valuable biomarkers, combined with clinical factors, to aid clinicians in the early diagnosis of BPD.

## Introduction

Bronchopulmonary dysplasia (BPD) is a common chronic respiratory complication in preterm infants. With advancements in drug-assisted therapy and critical care, the survival rate of preterm infants has increased significantly, and the incidence of BPD has also shown an increasing trend [1]. The underlying pathogenesis of BPD is multifactorial and results from the combined action of various factors that affect the immature lungs of premature infants, including genetic predisposition, infection, mechanical ventilation, hyperoxia exposure, and chemical stimulation [2]. Furthermore, there are currently no specific treatments available for BPD. Surviving children may experience persistent chronic respiratory dysfunction and neurological developmental disorders, significantly affecting their long-term quality of life and burdening families and society more [3]. Therefore, it is crucial to identify early predictive indicators of BPD and improve its outcome.

Mitochondria are essential organelles that provide energy, regulate immunity and signal transduction, and are closely associated with oxidative stress, immune regulation, and alveolar formation in vivo [4]. Kandasamy et al. [5] found that preterm infants with BPD exhibited more severe mitochondrial dysfunction, reduced mitochondrial oxygen consumption, and increased reactive oxygen species (ROS) production in human umbilical vein endothelial cells. Large amounts of ROS can activate NF-κB and STAT1 to promote lipid peroxidation, protein oxidation, DNA damage, nitrosation, and nitro-modification of target molecules, causing extensive damage to cells and tissues, which may play a crucial role in the development of BPD [6]. In an animal study, after establishing a mouse model of BPD and intervention with vitamin D, it was observed that vitamin D decreased mitochondrial damage and inhibited the expression of mitochondrial apoptosis-related proteins such as caspase-3 and Bax, resulting in improved lung injury [7]. Premature infants have an immature mitochondrial function. External triggers such as oxidative stress and infection may cause mitochondrial dysfunction, further affecting the body’s energy supply, antioxidant capacity, vascular remodelling, and lung branching morphology and promoting BPD development [8, 9]. Therefore, this study aims to explore the clinical utility of biomarkers, including mitochondrial dysfunction, inflammation, and alveolar polarization disorders in BPD.

MED1 is a well-known hypoxia-sensitive transcriptional coactivator and has been extensively studied in various diseases, such as atherosclerosis, pulmonary hypertension, breast cancer, etc. It binds to transcription factors and RNA polymerase II to exert biological effects [10–12]. A mouse model of common carotid artery stenosis after ligation demonstrated that MED1 lacks the pathway to activate NF-κB and STAT1, which leads to ROS accumulation, vascular inflammatory damage, and proliferation and migration of vascular smooth muscle cells [13]. Additionally, MED1 levels are lower in lung tissue or pulmonary artery endothelial cells in patients with idiopathic pulmonary hypertension and rodents with pulmonary hypertension. MED1 can interact synergistically with Kruppel-like factor 4 to maintain homeostasis in lung endothelial cells [10]. Furthermore, MED1 promotes mitochondrial biogenesis and oxidative phosphorylation.

As another essential transcriptional coactivator, PGC-1α primarily regulates various downstream pathways, including mitochondrial biogenesis, ROS detoxification, and oxidative phosphorylation, to play a crucial role in maintaining normal cell metabolism [14]. PGC-1α has been extensively studied in various conditions, including infectious, hyperoxygenetaion, aging, and apoptosis-related lung injury [15–17]. Yadav et al. [18] demonstrated that AMP-activated protein kinase could improve mitochondrial biogenesis and attenuate hyperoxic lung injury in rat pups by activating PGC-1α and its downstream pathways. In vitro cell experiments have shown that resveratrol, a natural agonist of exogenous NAD+-dependent protein deacetylase, can reduce ROS production, alleviate the decline of mitochondrial membrane potential through PGC-1α-related pathways, and improve hyperoxia-induced damage and apoptosis of human alveolar epithelial cells [19]. MED1 co-localizes with peroxisome proliferator-activated receptor-gamma protein and enhances its transcriptional activity [11]. MED1 may also mediate for PGC-1α-mediated production of antioxidant enzymes [13]. Therefore, this study aimed to analyze perinatal clinical data, serum MED1 and PGC-1α levels and explore their predictive value in BPD to establish a more comprehensive theoretical foundation for early prevention and treatment of BPD.

## Methods

### Patients

This prospective observational study was conducted at the Department of Neonatology, Children’s Hospital of Soochow University, from November 1, 2021, to November 1, 2022. Preterm infants with gestational age < 32 weeks and survival time ≥ 14 days admitted to the hospital within 24 h of birth were selected as study subjects. The BPD group comprised preterm infants who met the diagnostic criteria for BPD. The non-BPD group included randomly selected preterm infants who did not meet the diagnostic criteria for BPD, matched in a 1:1 ratio based on general data, including gender, gestational age, birth weight, etc. Maternal profiles were developed through standardized face-to-face interviews. Infants with chromosomal abnormalities or inherited metabolic disorders and those with incomplete clinical data were excluded. This study has been approved by the Ethics Committee of the Children’s Hospital of Soochow University (Ethics number: 2022CS135), and written informed consent was obtained from all parents of the infants.

### Definitions

The National Institute of Child Health and Human Development (NICHD) revised the diagnostic and grading criteria for BPD in 2018.BPD is diagnosed in preterm infants with imaging evidence of persistent parenchymal lung disease at gestational age < 32 weeks and who require continuous respiratory support for at least 3 days to maintain 90–95% oxygen saturation at 36 weeks of postmenstrual age (PMA) [20].

### Clinical variables

This study collected and analyzed clinical data from preterm infants and their pregnant mothers who met the admission criteria. The general conditions of preterm infants included gender, gestational age, birth weight, mode of delivery, whether in vitro fertilization was performed, the incidence of Apgar score ≤ 7 (1–5 min), and whether twin or multiple fetuses were present. General maternal pregnancy status included maternal age, gestational diabetes mellitus, gestational hypertension, pre-eclampsia, antenatal fever, preterm premature rupture of membrane ≥ 18 h, and use of medication (steroids, magnesium sulphate, antibiotics) before delivery. Primary underlying conditions and comorbidities in preterm infants included neonatal respiratory distress syndrome(NRDS), frequent apnea, severe asphyxia, pneumorrhagia, extrauterine growth restriction(EUGR), necrotizing enterocolitis(NEC), retinopathy of prematurity(ROP), ventilator-associated pneumonia(VAP), periventricular or intraventricular hemorrhage(PVH-IVH), parenteral nutrition-associated cholestasis(PNAC), patent ductus arteriosus(PDA), hemodynamic significant patent ductus arteriosus(hsPDA), sepsis, and bacterial meningitis. Laboratory findings within 24 h of admission included white blood cell count, red blood cell count, lymphocyte count, neutrophil count, platelet count, hemoglobin level, hematocrit, C-reactive protein, total protein, albumin, alanine transaminase, aspartate transaminase, aspartate transaminase/alanine transaminase ratio, glycocholic acid, fibrinogen, d-dimer, d-dimer/albumin ratio(DAR), and fibrinogen/albumin ratio(FAR). Treatment during hospitalization included the proportion and duration of invasive ventilation, the percentage of time on mechanical ventilation > 7 days, the days of oxygen inhalation, the duration to achieve total enteral nutrition, the percentage of infants on the time to achievetotal enteral nutrition for greater than 30 days, the number of red blood cell transfusions, the volume of red blood cell transfusions, and the use of caffeine, albumin, intravenous immunoglobulin, and fibrinogen.

### Serum biomarker collection and determination

One millilitre (1 ml) of peripheral venous blood was collected at postnatal days 1, 7, 14, 28 and PMA 36 weeks and centrifuged at 200 × g for 5 min using a Thermo Sorvall ST16R centrifuge. The upper serum were analyzed using enzyme-linked immunosorbent assay (ELISA) to determine the concentration of MED1 and PGC-1α in serum. PGC-1α assay kits were purchased from Saipason Biotechnology Co.,Shanghai, China, with product numbers SPS-12,473. MED1 assay kits were purchased from Ripan Biotechnology Co., Shanghai, China, with product numbers RE3232.

### Statistical analysis

Data analysis in this study was conducted using SPSS 26.0. Enumeration data were presented as cases (%) and analyzed using the chi-square test or Fisher’s exact probability method. Normally distributed measurement data were expressed as mean ± standard deviation, while non-normally distributed data were shown as median (25th percentile, 75th percentile), i.e., M(P25, P75). When data met normality and homogeneity of variance assumptions, T-tests were used for two-group comparisons, and analysis of variance was utilized for multiple-group comparisons. However, when data did not meet normality or homogeneity of variance assumptions, the Mann-Whitney U test was used to analyze two groups. In contrast, the Kruskal-Wallis H test was used for multiple groups comparison. Continuous variables were also analyzed by Pearson correlation analysis, yielding a correlation coefficient of *r*. All tests were two-sided, with statistical significance set at *P* < 0.05. Univariate analysis variables with *P* < 0.05 were subjected to multivariate regression analysis to explore postnatal risk factors related to BPD. The receiver operator characteristic(ROC) curve was used to evaluate the predictive efficiency of each index, and a logistic regression model was used to construct an early prediction model for BPD.

## Results

### Demographics of preterm infants

**Fig. 1:**
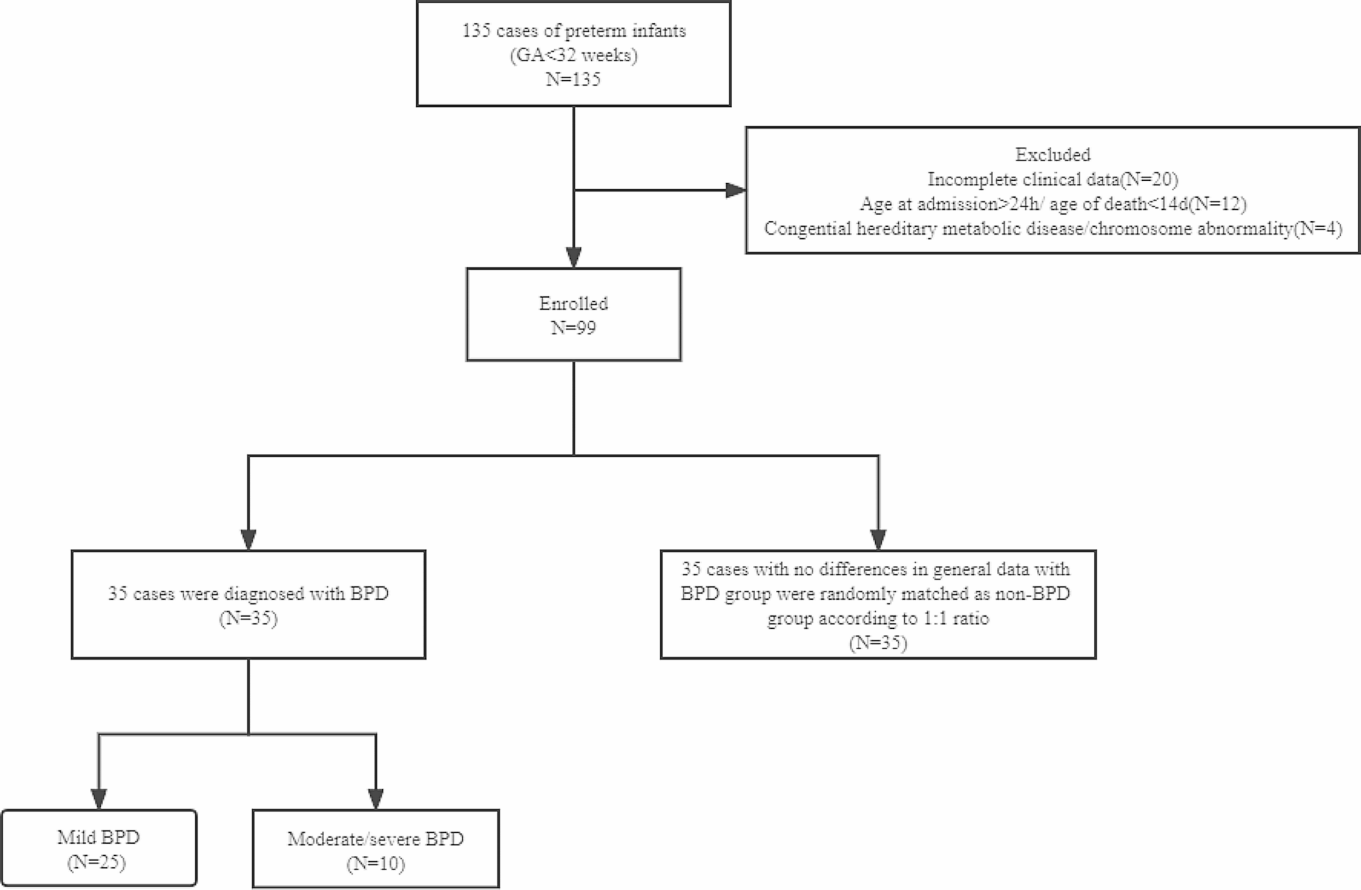
Flow chart of the research

Between November 01, 2021, and November 01, 2022, our hospital admitted 135 preterm infants under 32 weeks of gestational age. Exclusion criteria included incomplete clinical data (20 cases), genetic metabolic disease/chromosomal abnormalities (4 patients), age at admission > 24 h, and death within 14 days of admission (12 cases). Ultimately, 99 preterm infants were included in the study. Of these, 35 infants developed bronchopulmonary dysplasia (BPD), with 25 patients classified as mild BPD and 10 cases classified as moderate or severe BPD. 35 infants with general information such as sex, gestational age and birth weight matching the BPD group were randomly selected as the non-BPD group according to the 1:1 ratio (Fig. 1). There were no significant differences in baseline characteristics of infants and their pregnant mothers between the BPD and non-BPD group(*P* > 0.05) (Table 1), including gestational age, birth weight, sex, mode of delivery, test-tube baby or not, the proportion of Apgar score ≤ 7 (1–5 min), the proportion of maternal age < 20 or > 35 years, history of maternal pregnancy diseases (diabetes, pre-eclampsia, hypertension), history of delivery abnormalities (prenatal fever, preterm premature rupture of membranes ≥ 18 h), and history of prenatal medication (steroids, magnesium sulphate, antibiotics).

**Table 1:** Maternal and neonatal clinical characteristics

	BPD	Non-BPD	*P* Value
N (max)	35	35	
Male	16 (45.71%)	17 (48.57%)	0.811
Birthweight (g)	1245.14 (202.59)	1347.71 (232.53)	0.053
Gestation (wks)	29.55 (1.21)	30.08 (1.22)	0.076
C-section	22 (62.86%)	23 (65.71%)	0.803
Test-tube baby	8 (22.86%)	5 (14.29%)	0.356
twins or multiplets	5 (14.29%)	6 (17.14%)	0.743
APGAR at 1-min or 5-min ≤ 7 points	8 (22.86%)	5 (14.29%)	0.356
Maternal age < 20 or > 35 years	10 (28.57%)	6 (17.14%)	0.255
Antenatal fever	3 (8.57%)	1 (2.86%)	0.607
PPROM ≥ 18 h	8 (22.86%)	8 (22.86%)	1.000
Gestational diabetes mellitus	8 (22.86%)	9 (25.71%)	0.790
Pre-eclampsia	7 (20.00%)	8 (22.86%)	0.771
Gestational hypertension	4 (11.43%)	4 (11.43%)	1.000
Antenatal steroids	28 (80.00%)	30 (85.71%)	0.526
Antenatal magnesium sulphate	5 (14.29%)	2 (5.71%)	0.426
Antenatal antibiotics	6 (17.14%)	5 (14.29%)	0.743
White blood cells (10^9^/L)	8.94 (5.73,12.14)	8.01 (6.13,12.98)	0.911
Red blood cells (10^12^/L)	4.18 (0.60)	4.62 (0.56)	0.002
Lymphocyte (10^9^/L)	2.58 (1.77,3.74)	3.17 (2.18,3.67)	0.229
Neutrophils (10^9^/L)	4.84 (2.19,8.57)	3.84 (2.64,7.79)	0.856
Blood platelets (10^9^/L)	224.89 (65.51)	235.60 (67.53)	0.503
Hemoglobin content (g/L)	163.17 (22.95)	171.46 (22.79)	0.134
Hematocrit (L/L)	0.50 (0.07)	0.52 (0.06)	0.094
C-reactive protein > 8 mg/L	2 (5.71%)	0 (0.00%)	0.473
Serum albumin (g/L)	30.88 (5.02)	32.97 (2.84)	0.046
Serum total protein (g/L)	44.02 (8.30)	46.09 (4.92)	0.210
Serum alanine transaminase (U/L)	3.70 (2.70,5.10)	4.30 (3.00,6.90)	0.094
Serum aspartate transaminase (U/L)	50.90 (29.90,78.10)	39.40 (32.90,60.80)	0.518
Aspartate transaminase/alanine transaminase	13.11 (8.82,23.36)	9.25 (5.70,14.35)	0.033
Serum glycocholic acid (g/mL)	6.10 (3.23,13.96)	8.39 (3.96,12.23)	0.492
Fibrinogen (g/L)	1.24 (0.82,2.13)	1.23 (0.95,1.88)	0.522
D-dimer (µg/L)	4020.00 (1730.00,6220.00)	2170.00 (1370.00,5100.00)	0.066
DAR (µg/g)	131.75 (63.14,181.79)	65.83 (42.28,174.06)	0.044
FAR	0.04(0.03,0.08)	0.04(0.03,0.06)	0.288
Caffeine	31 (88.57%)	23 (65.71%)	0.023
Albumin	24 (68.57%)	8 (22.86%)	0.000
Intravenous immunoglobulin	27 (77.14%)	8 (22.86%)	0.000
Fibrinogen	1 (2.86%)	0 (0.00%)	1.000
Red blood cell infusion frequency	5.00 (4.00,6.00)	0.00 (0.00,2.00)	0.000
Red blood cell infusion (ml)	125.00 (101.00,154.00)	0.00 (0.00.60.00)	0.000
Days of oxygen inhalation (d)	57.00 (43.00,60.00)	29.00 (20.00,39.00)	0.000
Duration of invasive ventilation (d)	0.00 (0.00,5.00)	0.00 (0.00,0.00)	0.021
Invasive ventilation	17 (48.57%)	7 (20.00%)	0.012
Mechanical ventilation time > 7d	27 (77.14%)	10 (28.57%)	0.000
Time to achieve total enteral nutrition (d)	35 (26,42)	20 (16,24)	0.000
Time to achieve total enteral nutrition > 30d	26 (74.29%)	6 (17.14%)	0.000
Duration of hospital stays (d)	68.00 (55.00,82.00)	48.00 (40.00,55.00)	0.000
NRDS	23 (65.71%)	18 (51.43%)	0.225
Frequent apnea	18 (51.43%)	6 (17.14%)	0.003
Severe asphyxia	4 (11.43%)	1 (2.86%)	0.353
Pneumorrhagia	2 (5.71%)	1 (2.86%)	1.000
EUGR	15 (42.86%)	9 (25.71%)	0.131
NEC	4 (11.43%)	2 (5.71%)	0.393
ROP	9 (25.71%)	3 (8.57%)	0.057
SGA	6 (17.14%)	1 (2.86%)	0.111
CAM	3 (8.57%)	1 (2.86%)	0.607
VAP	1 (2.86%)	0 (0.00%)	1.000
PVH-IVH	7 (20.00%)	3 (8.57%)	0.172
PNAC	7 (20.00%)	2 (5.71%)	0.074
PDA	34 (97.14%)	30 (85.71%)	0.200
hsPDA	17 (48.57%)	9 (25.71%)	0.048
Sepsis	8 (22.86%)	5 (14.29%)	0.356
Bacterial meningitis	1 (2.86%)	0 (0.00%)	1.000

Note: Data are demonstrated as the mean (SD), n (%), or median (25th percentile, 75th percentile); Apnea in preterm infants: a respiratory arrest lasting more than 20 s, accompanied by a heart rate of less than 100 beats per minute or the presence of cyanosis, oxygen desaturation, and hypotonia; Frequent apnea: more than 2 recurrent episodes of apnea in 1 h or more than 6 episodes in 12 h

Abbreviation: BPD, bronchopulmonary dysplasia; PPROM, preterm premature rupture of membrane; DAR, D-dimer/Albumin ratio; FAR, Fibrinogen/Albumin ratio; NRDS, neonatal respiratory distress syndrome; EUGR, extrauterine growth restriction; NEC, necrotizing enterocolitis; ROP, retinopathy of prematurity; CAM, chorioamnionitis; SGA, small for gestational age; VAP, ventilator-associated pneumonia; PVH-IVH, periventricular or intraventricular hemorrhage; PNAC, parenteral nutrition-associated cholestasis; PDA, patent ductus arteriosus; hsPDA, hemodynamic significant patent ductus arteriosus

### Risk factors for BPD in preterm infants

Within 24 h of admission, ancillary tests in the BPD group showed lower peripheral blood red blood cell count and albumin levels compared to the non-BPD group. The Aspartate transaminase/alanine transaminase ratio and DAR were also higher in the BPD group than in the non-BPD group (*P* < 0.05) (Table 1). In terms of treatment, the rate of use of caffeine, albumin, intravenous immunoglobulin, fibrinogen utilization, invasive ventilation, mechanical ventilation > 7 days, the total number of days on oxygen, proportion of time to achieve total enteral nutrition and > 30 days, number of red blood cell infusions, and volume of red blood cell infusions was all significantly higher in the BPD group than in the non-BPD group (*P* < 0.05) (Table 1). Additionally, the incidence of frequent apnea and hsPDA was significantly higher in the BPD group than in the non-BPD group (*P* < 0.05) (Table 1). Binary logistic regression analysis identified frequent apnea (OR = 16.459, 95% CI: 1.578-171.629), duration of mechanical ventilation > 7 days (OR = 12.812, 95% CI: 1.655–99.168), and time to achieve total enteral nutrition (OR = 1.317, 95% CI: 1.110–1.561) as risk factors for BPD (*P* < 0.05).

### Serum MED1 levels

**Fig. 2:**
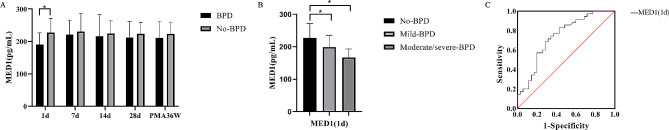
**(A)** Serum MED1 levels in BPD and non-BPD groups; **(B)** Serum MED1 levels in non-BPD, mild BPD, and moderate/severe subgroups; **(C)** ROC curve of serum MED1 level at the first day of life

- Serum MED1 levels in preterm infants in the BPD group were lower than those in the non-BPD group on the 1st, 7th, 14th, and 28th days of life and PMA 36 weeks, with a statistically significant difference in MED1 levels on the 1st day of life(*P* < 0.05) (Fig. 2A). MED1 levels on the 1st day of life were lower in the mild and moderate/severe BPD groups than in the non-BPD group (*P* < 0.05) (Fig. 2B). ROC curves were plotted with whether BPD occurred as the dependent variable and the MED1 level on the 1st day of life as the independent variable. The AUC for MED1 level on the 1st day of life predicting BPD was 0.740 (95% CI: 0.624–0.856, *P* < 0.05), with a critical value of 214.4 pg/mL and sensitivity and specificity of 77.1% and 62.9%, respectively (Fig. 2C).

### Serum PGC-1α levels

**Fig. 3:**
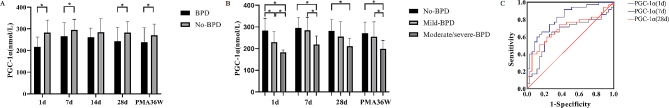
**(A)** Serum PGC-1α levels in BPD and non-BPD groups; **(B)** Serum PGC-1α levels in non-BPD, mild BPD, and moderate/severe subgroups(Fig. 3B); **(C)** ROC curve of serum PGC-1α level at the first day of life

Serum PGC-1α levels were lower in the BPD group than in the non-BPD group on the 1st, 7th, 14th, and 28th days of life and PMA 36 weeks, with statistically significant differences in PGC-1α levels on the 1st, 7th, 28th days of life and PMA 36 weeks(*P* < 0.05) (Fig. 3A). PGC-1α levels on the 1st and 7th days of life and at 36 weeks PMA were higher in the mild and non-BPD groups than in the moderate/severe BPD group (*P* < 0.05). Non-BPD infants also showed higher PGC-1α levels on the first day after birth compared to the mild BPD group, with levels on day 28 higher than those in the moderate/severe BPD group (*P* < 0.05) (Fig. 3B). The ROC curves were plotted using serum PGC-1α levels on the 1st, 7th, and 28th days of life as the independent variable and whether BPD occurred as the dependent variable. The AUCs for the ROC curves were 0.823 (95% CI: 0.727–0.919, *P* < 0.05), 0.649 (95% CI: 0.515–0.783, *P* < 0.05), and 0.698 (95% CI: 0.571–0.825, *P* < 0.05) with critical values of 240.7 nmol/L, 273.9 nmol/L, and 269.2 nmol/L, respectively. The sensitivity and specificity for the ROC curves were 74.3% and 74.3%, 62.9% and 74.3%, and 74.3% and 62.9%, respectively (Fig. 3C).

### Relevant analysis of MED1 and PGC-1 α level

Pearson correlation analysis was used to show that serum MED1 on the 1st, and 7th days of life was positively correlated with serum PGC-1 α levels, with correlation coefficient R-values of 0.882 and 0.693 (*P* < 0.05) (Table 2A), respectively. In addition, in the BPD group, serum MED1 was positively correlated with serum PGC-1 α levels on the 1st, 7th, and 14th days of life, with correlation coefficient R-values of 0.865, 0.916, and 0.829(*P* < 0.05) (Table 2B), respectively. In the non-BPD group, serum MED1 was positively correlated with serum PGC-1 α levels on the 1st, 7th, and 28th days of life and PMA 36 weeks, with correlation coefficient R-values of 0.849, 0.509, 0.403, and 0.464 (*P* < 0.05) (Table 2C), respectively.

**Table 2A:** Relevant analysis of MED1 and PGC-1 α level

Pearson correlation analysis		PGC-1 α(1d)	PGC-1 α(7d)	PGC-1 α(14d)	PGC-1 α(28d)	PGC-1 α(PMA36W)
MED1 level at the same time as PGC-1 α	*r*	0.882	0.693	0.107	0.104	0.174
	*P*	0.000	0.000	0.378	0.394	0.151
	N	70	70	70	70	70

Abbreviation: MED1, Mediator complex subunit 1; PGC-1α, Peroxisome proliferator-activated receptor gamma coactivator-1alpha

**Table 2B:** Relevant analysis of MED1 and PGC-1 α level in BPD group

Pearson correlation analysis		PGC-1 α(1d)	PGC-1 α(7d)	PGC-1 α(14d)	PGC-1 α(28d)	PGC-1 α(PMA36W)
MED1 level at the same time as PGC-1 α	*r*	0.865	0.916	0.829	-0.130	-0.037
	*P*	0.000	0.000	0.015	0.457	0.831
	N	35	35	35	35	35

Abbreviation: BPD, bronchopulmonary dysplasia; MED1, Mediator complex subunit 1; PGC-1α, Peroxisome proliferator-activated receptor gamma coactivator-1alpha

**Table 2C:** Relevant analysis of MED1 and PGC-1 α level in non-BPD group

Pearson correlation analysis		PGC-1 α(1d)	PGC-1 α(7d)	PGC-1 α(14d)	PGC-1 α(28d)	PGC-1 α(PMA36W)
MED1 level at the same time as PGC-1 α	*r*	0.849	0.509	-0.027	0.403	0.464
	*P*	0.000	0.002	0.880	0.016	0.005
	N	35	35	35	35	35

Abbreviation: BPD, bronchopulmonary dysplasia; MED1, Mediator complex subunit 1; PGC-1α, Peroxisome proliferator-activated receptor gamma coactivator-1alpha

### Predictive efficacy of risk factors for BPD

**Fig. 4:**
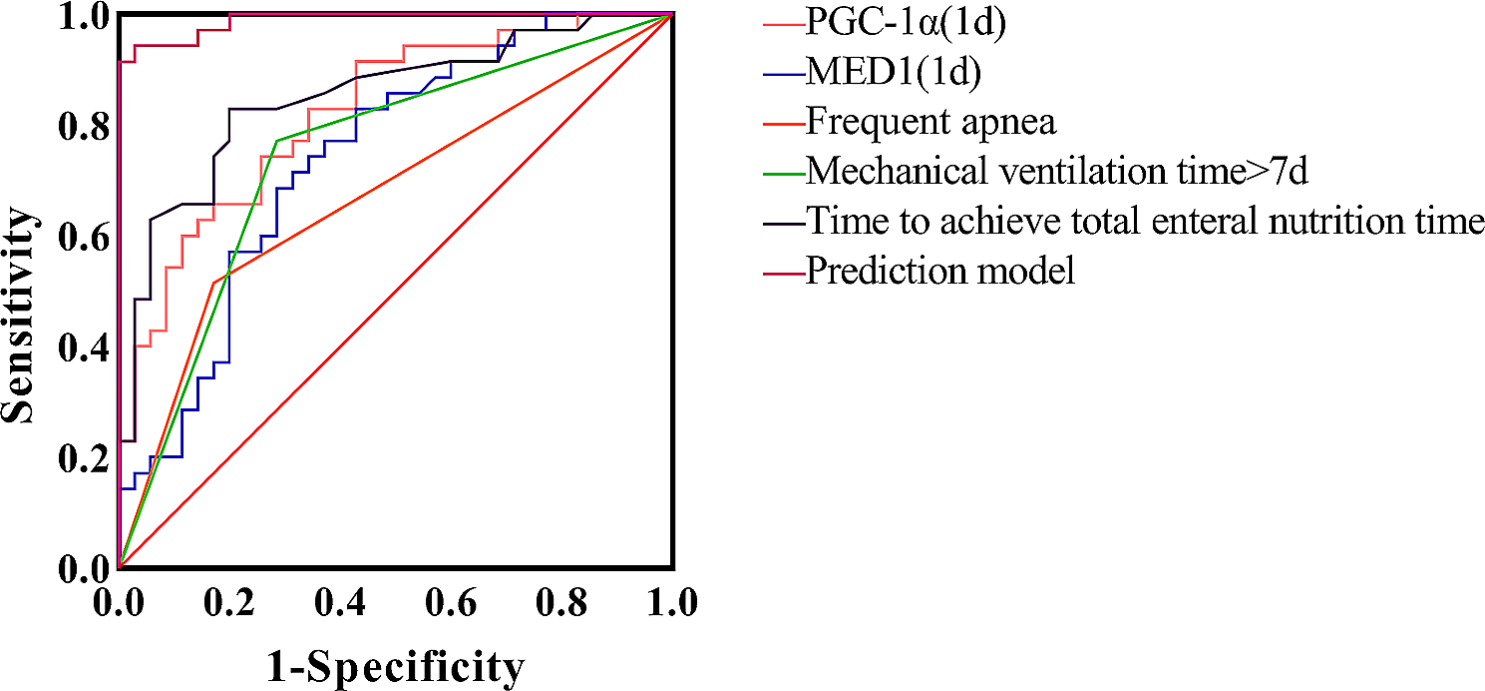
The total predictive effectiveness of BPD.

ROC curves were drawn using five variables: frequent apnea, duration of mechanical ventilation > 7 days, time to achieve total enteral nutrition, serum MED1 level on the 1st day of life and serum PGC-1α level on the 1st day of life. The efficacy of each of these indicators in predicting the occurrence of BPD was statistically significant when compared to the non-BPD group(*P* < 0.05) (Fig. 4).

### BPD prediction model

The BPD prediction model was constructed using five indicators: frequent apnea, time on mechanical ventilation > 7 days, time to achieve total enteral nutrition, serum MED1 level on the 1st day of life, and serum PGC-1α level on the 1st day of life. The regression equation for the model is *P* = 1/[1 + e-(11.524 + 4.931 × 1 + 3.829 × 2 + 0.278 × 3-0.051 × 4-0.046 × 5)], where X1, X2, X3, X4, and X5 represent frequent apnea, time on mechanical ventilation > 7 days, time to achieve total enteral nutrition, serum MED1 level on the 1st day of life, and serum PGC-1α level on the 1st day of life, respectively. *P* represents the logistic model’s predicted probability. The chi-square value of the model was 76.804 (*P* < 0.05), indicating that each of the above indicators has a predictive value for BPD in preterm infants. The Hosmer-Lemeshow was calculated using a solid interaction table, df = 8, *P* > 0.05, indicating a good fit for this predictive model.The area under the ROC curve (AUC) of the combined logistic regression model for the five predictors was 0.989, higher than the above indicators’ prediction alone (Table 3).

**Table 3:** Logistic regression for predicting BPD

Predictor	*P*	Odds ratio estimate	95% Confidence interval
Frequent apnea			
Yes	0.021	138.568	2.112∼9089.584
No		Reference	
Mechanical ventilation time > 7d			
Yes	0.015	46.024	2.105∼1006.068
No		Reference	
Time to achieve total enteral nutrition	0.004	1.321	1.095∼1.593
Serum MED1(1d)	0.029	0.950	0.907∼0.995
Serum PGC-1α(1d)	0.015	0.955	0.921∼0.991

Abbreviation: BPD, bronchopulmonary dysplasia; MED1, Mediator complex subunit 1; PGC-1α, Peroxisome proliferator-activated receptor gamma coactivator-1alpha

## Discussion

BPD is a common chronic lung disease in preterm infants. Its occurrence is closely related to various prenatal, intrapartum, and postnatal factors, such as the immaturity of lung tissue development, inflammation-induced lung tissue damage, and abnormal repair after injury [21]. In this prospective study, the postnatal risk factors for the development of BPD in preterm infants were analyzed by matching preterm infants with gestational age ≤ 32 weeks and their general maternal condition. We found that frequent apnea, mechanical ventilation > 7 days, and time to reach total enteral nutrition were significant risk factors for BPD in the postnatal period.

Children with BPD are primarily observed in ultra-immature preterm infants, whose lungs are still in the tubular and capsular stages of development at birth, functionally immature, and require different ventilation strategies to maintain early vital activities [22]. Exposure to continuous hyperoxia promotes ROS formation and triggers an oxidative stress response, resulting in excessive apoptosis of alveolar cells and affecting lung tissue growth, maturation, and repair [23–25]. Oxidative stress also disrupts mesenchymal-epithelial signalling, promotes the differentiation of alveolar adipose fibroblasts into myofibroblasts, inhibits the growth and differentiation of lung epithelial cells, and hinders the alveolarization process [26, 27].

MED1 is a critical transcriptional cofactor that regulates signal transduction by interacting with various nuclear receptors, including PPAR, ER, TR, GR, and RXR. It plays a crucial role in maintaining mitochondrial function and energy metabolism in the body [28]. Devor et al. [29] investigated placental tissues from pregnant women with pre-eclampsia. They found that the downregulation of Placenta-Specific Protein 1 expression might be related to hypoxia-sensitive transcriptional cofactor MED1 driving the corresponding program. Other studies have suggested that decreased MED1 expression levels were present in rodent models of pulmonary hypertension and lung endothelial cells, and overexpression of MED1 attenuated the pulmonary hypertensive phenotype in rodents, thereby promoting homeostasis of lung endothelial cells [10]. Our present study identified that MED1 levels on the 1st day of life were significantly lower in the BPD group than in the non-BPD group, and the AUC for BPD predicted by serum MED1 levels on the 1st day of life was 0.740. The depletion of MED1 in the early stages of BPD may lead to impaired pulmonary vascular endothelial stability and reduction in normal mitochondrial function, promoting BPD and forming a vicious circle.

PGC-1α, a major transcriptional co-activator involved in regulating mitochondrial biogenesis, anti-inflammation, and anti-oxidative stress, plays a critical role in maintaining normal metabolism in the body [14]. A related study showed that PGC-1α effectively reduced the expression of cyclooxygenase-2, inducible nitric oxide synthase, and their products and played an anti-inflammatory role in acute lung injury by constructing a mouse model [30]. Moreover, PGC-1α can reduce oxidative stress damage by inducing various ROS-detoxifying enzymes. The deficiency of PGC-1α increases susceptibility to oxidative stress [31]. In our study, serum PGC-1α levels were significantly lower in the BPD group than in the non-BPD group on the 1st, 7th, and 28th days of life and PMA 36 weeks. The predictive significance of serum PGC-1α was remarkably high on the 1st day of life, with an AUC of 0.823 for the ROC curve analysis. This finding may be due to the higher degree of oxidative stress in children with BPD due to early mechanical ventilation and infections, among others. The lower PGC-1α levels indicate poorer antioxidant and anti-inflammatory capacity, further contributing to the development and worsening of BPD.

Nascimento et al. [32] identified that the duration of invasive mechanical ventilation within 48 h after birth was a significant predictor of BPD. Our study findings were consistent with their results, where the proportion and duration of invasive mechanical ventilation, total oxygen duration, and mechanical ventilation > 7 days were higher in the BPD group than in the non-BPD group. The proportion of mechanical ventilation > 7 days was found to be a risk factor for BPD development in preterm infants, which is consistent with previous studies [33]. We also found frequent apnea was a risk factor for BPD development. This finding might be related to the immature lung tissues and respiratory centres in preterm infants with younger gestational age and lower birth weight, leading to an increased likelihood of apnea. Frequent apnea makes it difficult to withdraw from mechanical ventilation and prolongs the duration and frequency of oxygen administration, ultimately affecting lung tissue maturation [34, 35]. Protective ventilation strategies should be implemented while reducing invasive mechanical ventilation and shortening the duration of oxygen administration to mitigate lung oxidative stress and reduce the incidence of BPD.

Nutritional status is a crucial factor that affects the average growth and development of the body’s systems, organs, and tissues. In children with BPD, the risk of malnutrition is higher [36]. Malnutrition can contribute to the development of BPD by reducing lung weight, lung volume, elastic fibres, the number of cilia, and disturbing alveolar formation [37, 38]. Currently, most very premature infants require parenteral nutrition in the early postnatal period to meet the growing needs of the organism. A retrospective study analyzed the nutritional status of 564 preterm infants during the first 28 days of life. It revealed that children with BPD had a longer time to reach total enteral and parenteral nutrition. Multifactorial regression analysis showed that the time to achieve total enteral and parenteral nutrition were risk factors for malnutrition in BPD [39]. Our study suggests that the incidence of complete enteral nutrition beyond 30 days and the time taken to achieve total enteral nutrition are higher in the BPD group than in the non-BPD group. The duration of time required to reach total enteral nutrition is a risk factor for BPD development, which may be linked to the increased energy requirements of the organism, such as inflammation, infection, and growth in children with BPD, and the immature function of the gastrointestinal tract [40]. Reasonable nutritional strategies, along with early enteral feeding, may aid in decreasing the onset of BPD.

Various factors contribute to BPD, including immature lung development, mechanical ventilation, frequent apnea, and a prolonged time for total enteral nutrition. The changes in serum MED1 levels and PGC-1α levels are closely associated with BPD development. This study aims to construct a regression model by combining clinical risk factors and serological correlates to predict BPD occurrence in preterm infants better.

Several limitations exist in this study, including its single-centre design, small sample size, short duration, and absence of follow-up studies to assess post-discharge outcomes. Future multicenter, large-sample joint studies are necessary to validate the early diagnostic potential of MED1, PGC-1α, and BPD, optimize the model further, and enhance prediction accuracy.

## Conclusions

In conclusion, frequent apnea, mechanical ventilation > 7 days, and time to achieve total enteral nutrition were risk factors for BPD. Combining these clinical risk factors with serum MED1 and PGC-1α levels from the first day of life produced a regression model that enhanced the prediction accuracy of BPD in preterm infants.

## Data Availability

The datasets used and/or analysed during the current study are available from the corresponding author on reasonable request.
